# Supplementary figures and images for: Age-related response to mite parasitization and viral infection in the honey bee suggests a trade-off between growth and immunity
Source: PLoS One. 2023 Jul 17;18(7):e0288821. doi: 10.1371/journal.pone.0288821 (PMC10351714; doi:10.1371/journal.pone.0288821)

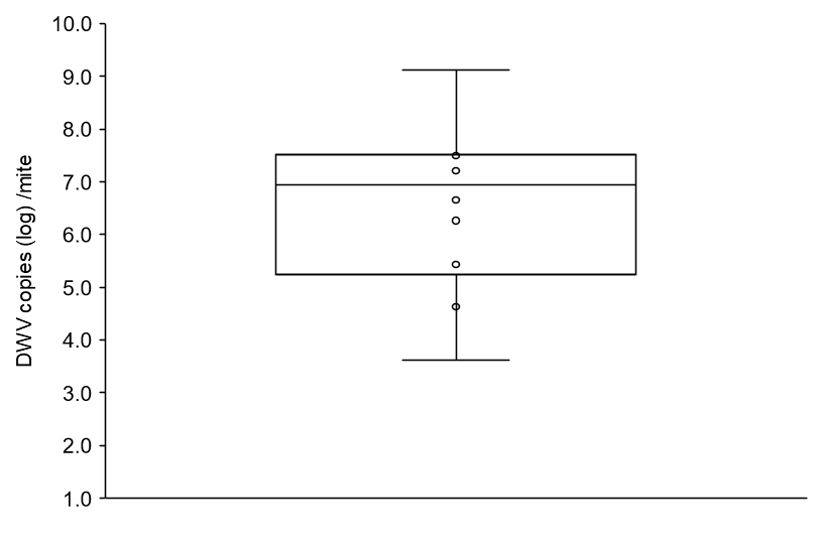


S1 Fig

Supplement: S1 Fig — Log transformed viral copy number in ten individual mites collected from the experimental apiary of the University of Udine during the period when the experiments were carried out. (DOCX) [file pone.0288821.s001.docx]
