# Supplementary material for: Age-related response to mite parasitization and viral infection in the honey bee suggests a trade-off between growth and immunity
Source: PLoS One. 2023 Jul 17;18(7):e0288821. doi: 10.1371/journal.pone.0288821 (PMC10351714; doi:10.1371/journal.pone.0288821)
Supplement: S1 Table — List of the genes differentially expressed between adult bees infested during the larval stage and uninfested. (DOCX) [file pone.0288821.s002.docx]

| **gff_id** | **gene name** | **padj** | **2FoldChange** |
| --- | --- | --- | --- |
| LOC412177 | 4-aminobutyrate aminotransferase, mitochondrial isoform X2 | 0.000 | -1.09095 |
| LOC409203 | UDP-glucuronosyltransferase 2A3 | 0.001 | 0.647377 |
| LOC726997 | uncharacterized protein LOC726997 | 0.001 | 0.863904 |
| LOC724471 | 1-phosphatidylinositol 4,5-bisphosphate phosphodiesterase-like | 0.001 | -1.00643 |
| LOC410915 | aquaporin AQPAn.G isoform X1 | 0.004 | 0.898363 |
| LOC409968 | protein transport protein Sec31A | 0.005 | 0.419029 |
| LOC408453 | cytochrome P450 9e2 | 0.005 | 0.919467 |
| LOC552531 | 10 kDa heat shock protein, mitochondrial | 0.007 | 0.888394 |
| LOC550791 | arrestin homolog | 0.007 | -0.94269 |
| LOC726850 | cytochrome b5 | 0.008 | 0.755716 |
| LOC412240 | DNA repair protein complementing XP-A cells homolog | 0.008 | -0.82883 |
| LOC552798 | putative sodium-coupled neutral amino acid transporter 10 isoform X2 | 0.011 | -0.63401 |
| LOC725348 | DNA mismatch repair protein Msh2 | 0.014 | -1.12492 |
| LOC551803 | homeotic protein spalt-major isoform X2 | 0.014 | 1.012067 |
| LOC412092 | myosin heavy chain, non-muscle isoform X3 | 0.016 | 0.734937 |
| LOC410527 | probable cation-transporting ATPase 13A3 isoform X2 | 0.017 | 0.973061 |
| LOC113218746 | jmjC domain-containing protein 5-like isoform X1 | 0.018 | -1.08694 |
| LOC552149 | aquaporin AQPAn.G isoform X1 | 0.020 | 0.918542 |
| LOC100576708 | recQ-mediated genome instability protein 1 | 0.021 | -1.13274 |
| LOC100576132 | uncharacterized protein LOC100576132 isoform X1 | 0.024 | -0.72271 |
| LOC113218929 | guanine nucleotide-binding protein subunit beta-like protein 1 | 0.024 | -0.71627 |
| LOC409475 | 26S proteasome non-ATPase regulatory subunit 2 | 0.024 | 0.430748 |
| LOC100578560 | 28S ribosomal protein S18c, mitochondrial | 0.024 | -0.9347 |
| LOC410784 | putative GTP-binding protein 6 | 0.028 | -0.75766 |
| LOC727398 | uncharacterized protein LOC727398 | 0.030 | -0.83183 |
| LOC551272 | talin-1 isoform X3 | 0.030 | 0.700032 |
| LOC100576242 | cilia- and flagella-associated protein 91 isoform X1 | 0.032 | -0.71052 |
| LOC552714 | conserved oligomeric Golgi complex subunit 2 | 0.032 | -0.79877 |
| LOC409675 | polyubiquitin-A isoform X2 | 0.033 | 0.416196 |
| LOC100577346 | sodium channel protein Nach | 0.033 | -1.51428 |
| LOC726711 | ribonuclease H2 subunit C | 0.035 | -1.6593 |
| LOC724480 | asparagine synthetase | 0.035 | -0.80197 |
| LOC726113 | protein-serine O-palmitoleoyltransferase porcupine | 0.035 | -1.43354 |
| LOC725344 | histone H2B | 0.036 | 0.910411 |
| LOC100577883 | cytochrome P450 4aa1-like isoform X1 | 0.036 | 1.896385 |
| LOC550749 | C-1-tetrahydrofolate synthase, cytoplasmic isoform X2 | 0.040 | 1.300263 |
| LOC409296 | T-complex protein 1 subunit gamma | 0.040 | 0.436725 |
| LOC725981 | protein TIPIN homolog | 0.042 | -1.42718 |
| LOC100577519 | alpha-(1,3)-fucosyltransferase 10, partial | 0.042 | -1.09346 |
| LOC102655737 | neural-cadherin isoform X6 | 0.042 | 0.857176 |
| LOC412778 | chondroitin sulfate proteoglycan 4 | 0.042 | 0.979572 |
| LOC551392 | U11/U12 small nuclear ribonucleoprotein 48 kDa protein | 0.042 | -0.98898 |
| LOC100576610 | mitochondrial ribonuclease P catalytic subunit isoform X1 | 0.042 | -0.76466 |
| LOC724974 | tubulin polyglutamylase TTLL7 | 0.042 | -1.23093 |
| LOC413400 | double-strand break repair protein MRE11 | 0.044 | -0.88339 |
| LOC726890 | carbonic anhydrase 2 | 0.048 | -1.34648 |
| LOC727193 | lipase member H-A | 0.050 | 3.461327 |

S1 Table
