# Supplementary material for: Age-related response to mite parasitization and viral infection in the honey bee suggests a trade-off between growth and immunity
Source: PLoS One. 2023 Jul 17;18(7):e0288821. doi: 10.1371/journal.pone.0288821 (PMC10351714; doi:10.1371/journal.pone.0288821)
Supplement: S2 Table — List of the genes differentially expressed between adult bees infested after the emergence and uninfested. (DOCX) [file pone.0288821.s003.docx]

| **gff_id** | **gene name** | **padj** | **2FoldChange** |
| --- | --- | --- | --- |
| LOC411577 | protein argonaute-2 | 0.022 | 1.255378 |
| LOC409306 | 2-oxoisovalerate dehydrogenase subunit beta, mitochondrial | 0.023 | 1.79091 |
| LOC100576458 | urea transporter 2 | 0.031 | 1.789072 |
| LOC411706 | phospholipase B1, membrane-associated | 0.044 | 1.288648 |

S2 Table
